# Combined Ultrasound-Guided Rectointercostal and Transversalis Fascia Plane Blocks for Postoperative Analgesia in a Pediatric Renal Transplant Recipient

**DOI:** 10.4274/TJAR.2026.262447

**Published:** 2026-08-28

**Authors:** Volkan Özen, Engin İhsan Turan, Bahadır Çiftçi, Selçuk Alver, Gökçen Şahin Şener, Ayça Sultan Şahin

**Affiliations:** 1 University of Health Science Türkiye, Prof. Dr. Cemil Taşçıoğlu City Hospital, Clinic of Anaesthesiology, İstanbul, Türkiye; 2 University of Health Science Türkiye, Kanuni Sultan Süleyman Training and Research Hospital, Clinic of Anaesthesiology, İstanbul, Türkiye; 3 İstanbul Medipol University Faculty of Medicine, Department of Anaesthesiology and Reanimation, İstanbul, Türkiye; 4 İstanbul Medipol University Faculty of Medicine, Department of Anatomy, İstanbul, Türkiye; 5 Biruni University Hospital, Clinic of Anaesthesiology and Reanimation, İstanbul, Türkiye

**Keywords:** Paediatric anaesthesia, pain, pain management, postoperative pain, regional anaesthesia

The optimization of postoperative analgesia for pediatric patients remains a critical challenge, particularly in complex surgical cases where systemic analgesic options are limited. Ultrasound-guided regional anaesthesia techniques have increasingly been adopted in children due to their opioid-sparing effects, favorable safety profile, and ability to provide targeted analgesia.^1^ Recently, the combination of multiple interfascial plane blocks has emerged as a promising strategy to enhance dermatomal coverage while minimizing systemic drug exposure.^2, 3, 4^ However, clinical data describing combined block applications in pediatric patients, especially those with significant comorbidities, remain limited.

A 3-year-old child weighing 12 kg underwent a living-donor renal transplantation with a total operative duration of 5 hours. Owing to the complexity of the procedure and the need for close postoperative monitoring, the patient was transferred to the pediatric intensive care unit while intubated and was extubated in the early postoperative period. Given the presence of underlying cirrhotic liver disease, an opioid-sparing multimodal analgesic strategy was preferred. The surgical incision extended across the abdominal wall, involving both upper and lower abdominal dermatomal territories. Because this pediatric patient’s renal transplantation required broad abdominal wall analgesia rather than analgesia limited to a single unilateral cutaneous distribution, a bilateral block strategy was selected. Bilateral rectointercostal plane blocks were intended to cover the upper anterior abdominal wall, whereas bilateral transversalis fascia plane blocks were intended to complement analgesia of the lower abdominal wall. This approach was chosen to provide broader, more continuous somatic analgesic coverage while maintaining an opioid-sparing strategy in a patient with limited systemic analgesic options. (Figure 1).

Written informed consent was obtained from the legal guardian of the patient for publication of the case details and images.

All blocks were performed under sterile conditions with the patient in the supine position. A high-frequency linear ultrasound transducer (6-13 MHz) was used.

For the rectointercostal plane block, the transducer was placed in a sagittal orientation just lateral to the midline at the level of the costal margin. The rectus abdominis muscle, costal cartilage, and underlying intercostal muscles were identified. Using an in-plane approach, a 22-gauge short-bevel needle was advanced cranial-to-caudal into the fascial plane between the posterior aspect of the rectus abdominis muscle and the costal cartilage and intercostal muscle complex.

For the transversalis fascia plane block, the transducer was positioned transversely along the mid-axillary line between the iliac crest and the costal margin. The external oblique, internal oblique, and transversus abdominis muscles were identified, and the transversalis fascia deep to the transversus abdominis muscle was visualized. Using an in-plane technique, the needle was advanced in a lateral-to-medial direction into the fascial plane between the transversus abdominis muscle and the transversalis fascia. After careful aspiration, hydrodissection confirmed correct placement and the local anaesthetic was administered with visible spread along the plane.

For local anaesthetic administration, the dose was calculated according to the patient’s body weight and distributed equally among the four injection sites. For each rectointercostal plane block, 1.25 mL of 0.5% bupivacaine, corresponding to 6.25 mg, was diluted with 4 mL of normal saline and then administered to each side. The same volume and concentration were used for each transversalis fascia plane block; therefore, 1.25 mL of 0.5% bupivacaine diluted with 4 mL of normal saline was administered on both the right and left sides. In total, four injections were performed: bilateral rectointercostal plane blocks and bilateral transversalis fascia plane blocks. The cumulative dose of bupivacaine was 25 mg, corresponding to approximately 2 mg kg^-1^, which was within the recommended safe dosing limits for pediatric patients.

Postoperative pain was assessed using the face, legs, activity, cry, consolability (FLACC) scale were performed by an anaesthesiologist experienced in pediatric pain evaluation, using a standardized assessment approach at predefined time points. Analgesia remained effective throughout the 24-hour follow-up period. The FLACC score was 0/10 at 30 minutes post-extubation, and remained low (1/10) during both the 1^st^ and 2^nd^ postoperative hours. At the 4^th^ postoperative hour, the FLACC score increased modestly to 2/10, at which point intravenous paracetamol (10 mg kg^-1^) was administered as rescue analgesia. Subsequent assessments demonstrated sustained pain control, with FLACC scores of 1/10 at the 6^th^ and 12^th^ postoperative hours. At 24 hours postoperatively, the FLACC score remained at 1/10. A second dose of intravenous paracetamol (10 mg kg^-1^) was administered during the first postoperative day.

From an anatomical perspective, the combination of rectointercostal and transversalis fascia plane blocks may provide complementary dermatomal coverage. The rectointercostal plane block primarily targets the anterior cutaneous branches of the upper intercostal nerves (approximately T6-T9), contributing to analgesia of the upper anterior abdominal wall. In contrast, the transversalis fascia plane block targets the lower thoracic and upper lumbar nerves, particularly T12-L1 (iliohypogastric and ilioinguinal nerves), thereby providing sensory coverage of the lower abdominal region. This combined approach may therefore achieve broader and more continuous analgesic coverage than single interfascial plane techniques.

Recent case reports have suggested that the combination of different interfascial plane blocks may result in wider dermatomal spread and more comprehensive analgesic coverage than single-block techniques.^2, 3, 4^ In line with these observations, the low and stable FLACC scores in our patient during both the early and late postoperative periods indicate that such a combined approach may provide effective postoperative analgesia, potentially covering both the anterior abdominal wall and deeper somatic pain components. However, these findings are limited to a single case and therefore remain hypothesis-generating. While our results support the feasibility and potential analgesic benefit of combined rectointercostal and transversalis fascia plane blocks, conclusions regarding efficacy, extent of spread, and clinical superiority cannot be drawn beyond the level of a case report.

Combined bilateral rectointercostal and transversalis fascia plane blocks may offer effective, opioid-sparing postoperative analgesia in pediatric patients with restricted systemic analgesic options and warrant further prospective evaluation.

## Figures and Tables

**Figure 1:**
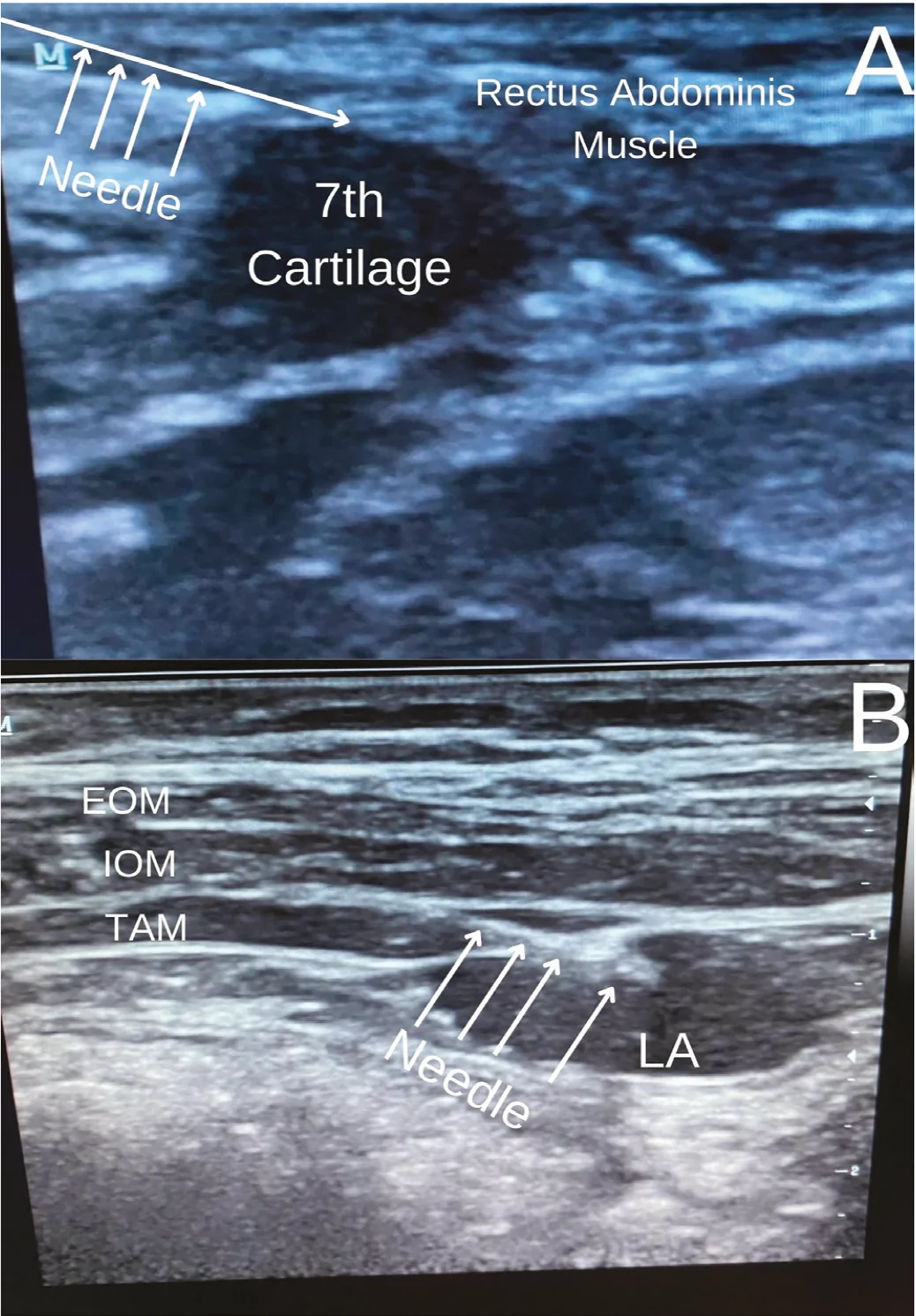
Applications of the blocks. A) Rectointercostal block. B) Transversalis fascia plane block. LA, local anaesthetic; EOM, external oblique muscle; IOM, internal oblique muscle; TAM, transversus abdominis muscle.
